# Brown adipose tissue and type 2 diabetes

**DOI:** 10.1093/emph/eoaa010

**Published:** 2020-04-06

**Authors:** Stephanie B Levy

**Affiliations:** e1 Department of Anthropology, CUNY Hunter College, New York, NY, USA; e2 New York Consortium in Evolutionary Primatology, New York, NY, USA

**Keywords:** energy expenditure, thermogenesis, blood pressure, adaptation

## Abstract

Recent work proposes that a regimen of repeated mild cold exposure may have protective effects against the development of type II diabetes mellitus (T2D) by activating brown adipose tissue (BAT) metabolism. BAT may protect against by increasing whole-body energy expenditure and insulin sensitivity. An evolutionary perspective, however, highlights several limitations of this hypothesis. Some individuals adapt to acute cold stress by constricting their blood vessels, which leads to high blood pressure. Thus, a regimen of repeated mild cooling may have beneficial health effects for some individuals and negative consequences for others. Future research should examine the relationships between low temperature exposure, BAT metabolism, blood pressure, and type II diabetes risk.

## TYPE II DIABETES

Recent work proposes that growing rates of obesity-related diseases, such as type II diabetes mellitus (T2D), may in part be due to reductions in cold-induced energy expenditure (EE) [1]. The social and economic conditions that underlie the global increase in high-calorie diets and sedentary lifestyles may also cause adults to spend more time in temperature-controlled spaces. This can restrict the number of calories an individual expends producing body heat, thus increasing the likelihood of gaining excess weight. Additionally, the metabolic pathways involved in thermogenesis appear to have a beneficial effect on insulin sensitivity [2].

In response to this idea, some researchers propose that a regimen of repeated cold exposure may prevent the development of T2D by activating brown adipose tissue (BAT) metabolism [2]. One way the body adapts to low temperatures is by triggering BAT deposits to convert stored energy into heat. This process, called BAT thermogenesis, not only generates an increase in EE, but also leads to improvements in glucose clearance and insulin sensitivity [1]. Although BAT thermogenesis may have protective effects against T2D; further research is required before clinical recommendations can be made.

## EVOLUTIONARY PERSPECTIVES

An evolutionary perspective reveals two critical limitations to the cold regimen treatment hypothesis. First, the body utilizes multiple mechanisms when adapting to low temperatures. Although some individuals rely heavily on metabolic adaptations to cold stress, others fail to exhibit cold-induced increases in EE and adapt primarily by preventing heat loss through vasoconstriction [3]. Thus, cold exposure may have protective effects against T2D risk for some individuals but increase the risk of high blood pressure in others depending on which adaptive mechanisms are triggered. Even mild cooling of 20°C can cause an increase in blood pressure. Furthermore, past work documents an association between cold-induced elevations in blood pressure and wintertime surges in cardiovascular disease morbidity and mortality [3].

Second, the degree to which an individual relies on BAT thermogenesis as an adaptation to cold stress may depend on the amount of cold exposure, they encountered early in life due to developmental plasticity in BAT. Data collected among the Yakut, an indigenous population in northeastern Siberia, found that, after a 30-min mild cooling treatment (15°C cooling suit), some participants exhibit an increase in BAT-associated EE of over 30%, while other exhibit a decline in EE of 20% [4]. Cold-induced declines in EE are likely due to vasoconstriction of the peripheral tissues, limiting oxygen transport and resulting in a net decline in whole body EE. Yakut participants that spent more time outside during early childhood exhibit greater BAT thermogenesis and cold-induced EE in adulthood, indicating that there may be developmental programming of BAT growth and metabolism.

## FUTURE IMPLICATIONS

A regimen of mild cooling may have beneficial effects for some adults (e.g. increasing glucose clearance) and negative consequences for others (e.g. raising blood pressure), depending on the degree to which an individual utilizes metabolic versus vasoconstrictive responses to cold stress. Although one study documents a negative association between cold-induced EE and blood pressure, more research is needed to investigate the relationships between BAT thermogenesis and vasoconstriction [3]. Additionally, future research should examine the relationship between exposure to low temperatures during development, BAT thermogenesis and T2D risk.

*Conflict of interest statement*. None declared.
